# Cellular Repair of DNA–DNA Cross-Links Induced by 1,2,3,4-Diepoxybutane

**DOI:** 10.3390/ijms18051086

**Published:** 2017-05-18

**Authors:** Lisa N. Chesner, Amanda Degner, Dewakar Sangaraju, Shira Yomtoubian, Susith Wickramaratne, Bhaskar Malayappan, Natalia Tretyakova, Colin Campbell

**Affiliations:** 1 Department of Pharmacology, University of Minnesota, Minneapolis, MN 55455, USA; ches0129@umn.edu (L.N.C.); syomtoubian@gmail.com (S.Y.); 2 Department of Medicinal Chemistry, University of Minnesota, Minneapolis, MN 55455, USA; degne013@umn.edu (A.D.); sanga016@umn.edu (D.S.); wickr011@umn.edu (S.W.); bhaskarmal@gmail.com (B.M.); trety001@umn.edu (N.T.)

**Keywords:** interstrand DNA-DNA crosslink, DNA repair, Fanconi anemia, nucleotide excision repair, homologous recombination, Chinese hamster lung fibroblast, 1,2,3,4-diepoxybutane, DNA double strand break

## Abstract

Xenobiotic-induced interstrand DNA–DNA cross-links (ICL) interfere with transcription and replication and can be converted to toxic DNA double strand breaks. In this work, we investigated cellular responses to 1,4-*bis*-(guan-7-yl)-2,3-butanediol (*bis*-N7G-BD) cross-links induced by 1,2,3,4-diepoxybutane (DEB). High pressure liquid chromatography electrospray ionization tandem mass spectrometry (HPLC-ESI^+^-MS/MS) assays were used to quantify the formation and repair of *bis*-N7G-BD cross-links in wild-type Chinese hamster lung fibroblasts (V79) and the corresponding isogenic clones V-H1 and V-H4, deficient in the *XPD* and *FANCA* genes, respectively. Both V-H1 and V-H4 cells exhibited enhanced sensitivity to DEB-induced cell death and elevated *bis*-N7G-BD cross-links. However, relatively modest increases of *bis*-N7G-BD adduct levels in V-H4 clones did not correlate with their hypersensitivity to DEB. Further, *bis*-N7G-BD levels were not elevated in DEB-treated human clones with defects in the *XPA* or *FANCD2* genes. Comet assays and γ-H2AX focus analyses conducted with hamster cells revealed that ICL removal was associated with chromosomal double strand break formation, and that these breaks persisted in V-H4 cells as compared to control cells. Our findings suggest that ICL repair in cells with defects in the Fanconi anemia repair pathway is associated with aberrant re-joining of repair-induced double strand breaks, potentially resulting in lethal chromosome rearrangements.

## 1. Introduction

The anticancer activity of many clinically useful drugs including platinum compounds, mitomycin C, psoralens, and antitumor nitrogen mustards is attributed to their ability to form chromosomal interstrand DNA–DNA cross-links (ICLs) [1]. ICLs can also be induced by endogenous lipid peroxidation products such as α, β-unsaturated aldehydes [2,3] and diepoxides such as the ultimate genotoxic metabolite of butadiene, 1,2,3,4-diepoxybutane, DEB [4]. If left unrepaired, ICLs prevent DNA strand separation required for normal replication and transcription [1,5], leading to selective toxicity in rapidly proliferating cells such as tumor cells [6,7,8,9].

Cellular ICL lesions present particular challenges to the DNA repair machinery because they inhibit local duplex melting at the site of adduct formation. Furthermore, both DNA strands at the site of the cross-link are damaged, potentially compromising their use as templates for accurate repair synthesis [1]. The mechanisms through which cells repair ICLs are a subject of active research. In *Escherichia coli*, elements of nucleotide excision repair (NER) and homologous recombinational repair (HR) pathways are believed to collaborate in ICL repair [10,11,12].

There is evidence that ICL repair in mammalian cells can occur via a process that is independent of recombination. For example, NER-dependent ICL repair has been observed in G1 arrested cells, although it is not clear whether this mechanism is restricted to this phase of the cell cycle [13,14]. In addition, it is noteworthy that ICLs can be converted to DNA double strand breaks (DSBs) in yeast [15,16,17] and in mammalian cells [18,19,20]—an outcome not observed in bacteria [12]. Considerable uncertainty remains regarding the precise mechanisms through which ICLs are converted to DSBs however, it appears likely that this process occurs when replication forks encounter ICL lesions during the S phase of the cell cycle [5,18,21]. The identity of the nuclease(s) involved in ICL removal is yet to be conclusively established, although *XPF-ERCC1*, *MUS81-EME1*, *SLX1-SLX4*, *FAN1*, *SNM1A*, and *SNM1B* have been implicated [22]. In addition, translesion synthesis polymerases have been proposed to contribute to ICL tolerance and repair in eukaryotes [12,23].

Fanconi anemia (FA) is a heterogeneous autosomal recessive disorder that predisposes individuals to cancer. Patients with FA and cells derived from FA patients display an enhanced sensitivity to DNA damaging agents that induce ICLs [24]. It was recently reported that biallelic mutations in the human *XPF* gene cause FA [25,26]. The *XPF* gene encodes a protein that forms a heterodimer with the *ERCC1* gene product and participates in NER by cleaving DNA on the 5′ side of helix-distorting lesions [27]. More typically, inactivation of NER genes in humans is associated with the disorder xeroderma pigmentosum. People with this disorder are predisposed to skin cancer, and cells derived from these individuals display hypersensitivity to ultraviolet radiation [27]. This previously unappreciated genetic connection between xeroderma pigmentosum and FA may help explain earlier observations that cells with defects in *XPF/ERCC1* are particularly sensitive to ICL-inducing agents [28], while clones with defects in other NER genes display a more modest sensitivity to these agents [29]. This further suggests that there is crosstalk between the two DNA repair pathways [30], and that a primary function of the FA pathway is to coordinate the cellular response to ICLs [1,30,31].

**Scheme 1:**
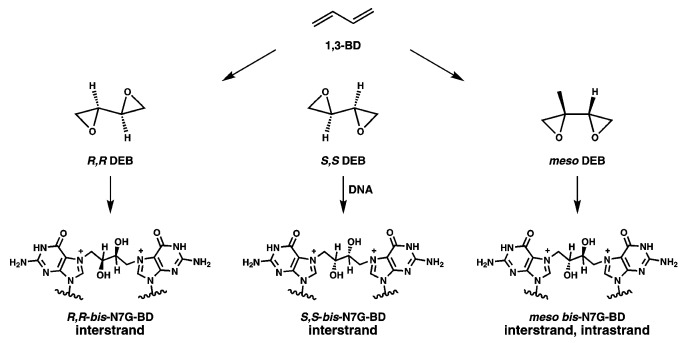
Metabolic activation of 1,3-butadiene to 1,2,3,4-diepoxybutane (DEB) and the induction of *bis*-N7G-BD DNA–DNA cross-links.

To explore the respective roles of NER and FA pathways in ICL repair, we examined the cellular responses of wild-type and repair-deficient cells to the DNA cross-linking agent 1,2,3,4-diepoxybutane (DEB). DEB is considered the ultimate carcinogenic species of 1,3-butadiene, a common environmental and industrial chemical present in cigarette smoke and urban air [32]. DEB is known to induce a variety of DNA lesions including nucleobase monoadducts, DNA-protein cross-links, and both intrastrand and interstrand DNA–DNA cross-links [33,34]. It sequentially alkylates guanine bases within DNA to form interstrand and intrastrand 1,4-*bis*-(guan-7-yl)-2,3-butanediol cross-links (*bis*-N7G-BD). Previous studies conducted in our laboratory have revealed that the cross-linking specificity of DEB is dependent on its stereochemistry: while *SS*- and *RR*- DEB preferentially form interstrand lesions, the *meso*- isomer generates equal numbers of intrastrand and interstrand lesions (Scheme 1) [4,35,36]. Both intrastrand and interstrand *bis*-N7G-BD lesions are hydrolytically labile, with average half-lives of 3.5–4 days under physiological conditions [37].

In the present work, we followed the formation and repair of DEB-induced *bis*-N7G-BD DNA–DNA cross-links and DNA double strand breaks in human and Chinese hamster fibroblast cell lines proficient or deficient in NER and FA recombination repair pathways. Our studies involved Chinese hamster fibroblasts (V79) and the isogenic derivative clones V-H1 and V-H4, deficient in the *XPD* and *FANCA* genes, respectively, as well as human cell lines with defects in the *XPA* and *FANCD2* genes. Furthermore, we examined cell viability and investigated changes in cell cycle dynamics in wild-type and repair-deficient clones following treatment with DEB.

## 2. Results

### 2.1. Cell Viability in the Presence of 1,2,3,4-Diepoxybutane (DEB)

**Figure 1:**
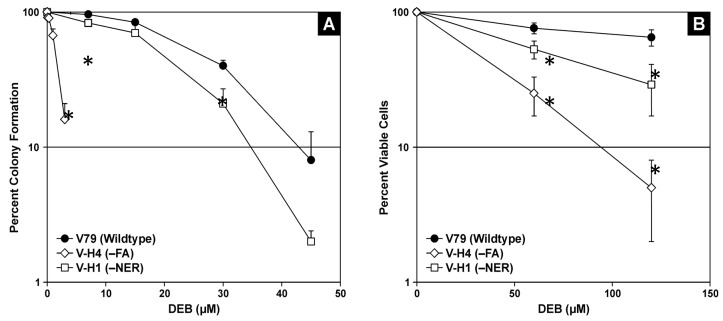
Effects of exposure to 1,2,3,4-diepoxybutane (DEB) on colony formation and survival of V79 (circles), V-H1 (squares) and V-H4 (diamonds) cells. The influence of exposure to various concentrations of DEB for 3 h on colony forming activity (**A**, clonogenic assay), and survival (**B**, direct cell counting of trypan blue-excluding cells) was determined as described in the Methods section. Results represent average ± the standard error of the mean (SEM), *n* = 3 or more. * *p* < 0.05.

To establish the effects of DEB exposure on cell viability, V79, V-H4, and V-H1 cells were treated with increasing concentrations of DEB in serum-free growth media for 3 h, and cellular DEB sensitivity was analyzed using a clonogenic assay [38]. As shown in Figure 1A, DEB exposure exerted a substantially greater inhibitory effect on colony formation in FA-deficient (V-H4) cells as compared to the parental V79 cell line. The concentration of DEB required to reduce colony formation by 50% (IC_50_) in V-H4 cells (1.4 µM) was ~18-fold lower than the IC_50_ in V79 cells (25 µM, Figure 1A). This result confirms that the V-H4 clone is hypersensitive to cell death induced by *bis*-electrophiles, probably due to a deficiency in the *FANCA* gene [39]. NER-deficient V-H1 cells also demonstrated increased sensitivity to DEB treatment (IC_50_ value, 19 µM) but were not as sensitive to DEB as the V-H4 clone (Figure 1A). The latter result is consistent with published reports of modest sensitivity of *XPD*-deficient Chinese hamster-derived clones towards other DNA–DNA cross-linking agents such as nitrogen mustards [18,29].

Xenobiotic-induced reductions in colony forming ability are generally interpreted to indicate cell death [38]. However, a substantial number of cells exposed to *bis*-electrophiles such as mitomycin C or DEB remain viable, yet permanently exit the cell cycle [40,41]. This phenomenon has been exploited to create non-dividing “feeder cells” used to support the cultivation of specialized cell lineages, including embryonic stem cells [42]. Therefore, it is likely that the reduction in colony formation associated with exposure to DEB, and the formation of ICLs over-estimates the acute cytotoxic effect of DEB exposure on V79, V-H1, and V-H4 cells. Therefore, we analyzed cellular viability in the presence of DEB directly by counting trypan blue-excluding cells 24 h post-treatment. The results from this analysis (Figure 1B) indicate that the concentration of DEB needed to reduce viable cell counts by 50% (LC_50_) in V-H4 cells (30 µM) was ~6 fold lower than the LC_50_ of DEB in V79 cells (165 µM). Parallel experiments revealed that the LC_50_ of DEB in V-H1 cells was ~2 fold lower than observed in V79 cells (Figure 1B). Taken together, the results from Figure 1 indicate that among the three cell lines examined, FA-deficient V-H4 cells exhibit the greatest sensitivity to DEB, followed by NER-deficient V-H1 clone and wild-type fibroblasts.

### 2.2. Effects of DEB Exposure on Cell Cycle Dynamics

**Figure 2:**
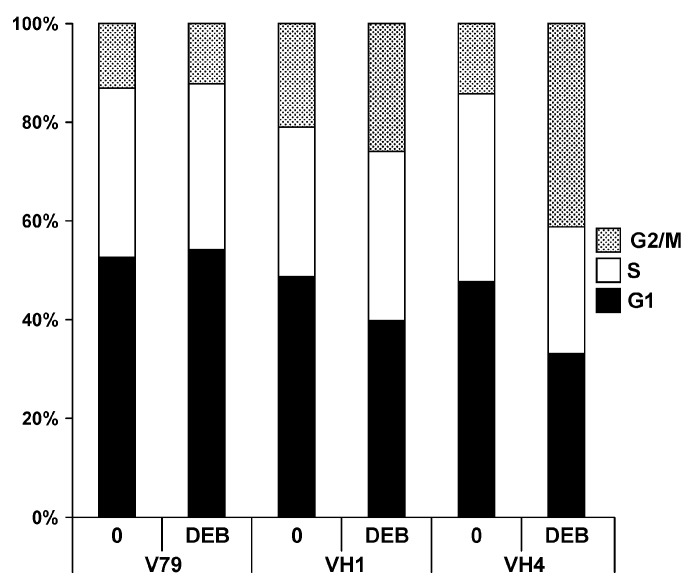
Cell cycle distribution of V79, V-H1 and V-H4 cells prior to and 24 h post exposure to DEB. Cells incubated for three hours in the absence (0) or presence (15) of 15 µM DEB were subjected to flow cytometry as described in the Methods section. The image depicts the percentage of cells in the G1, S and G2/M phases of the cell cycle. Results represent the average of three separate experiments. DEB exposure significantly increased, or decreased, respectively, the percentage of V-H4 cells in G2/M and S phases of the cell cycle. *p* < 0.05, *t*-test with Bonferroni correction.

To evaluate the effects of DEB treatment on cell cycle dynamics, V79, V-H1, and V-H4 cells were subjected to flow cytometry analyses 24 h post-exposure to normal media or media containing 15 μM DEB. These experiments have revealed that DEB treatment had little effect on cell cycle distribution in V79 and V-H1 cells (Figure 2). In contrast, DEB treatment of V-H4 cells resulted in a pronounced increase in the percentage of cells in G2/M phase, along with the corresponding decrease in the relative percentage of cells in the S phase of the cell cycle (Student *t* test, *p* < 0.01, Bonferroni correction). This finding indicates that FA-deficient cells display G2/M cell cycle arrest following exposure to DEB.

### 2.3. Bis-N7G-BD Adduct Formation in DEB-Treated Hamster Cells

**Figure 3:**
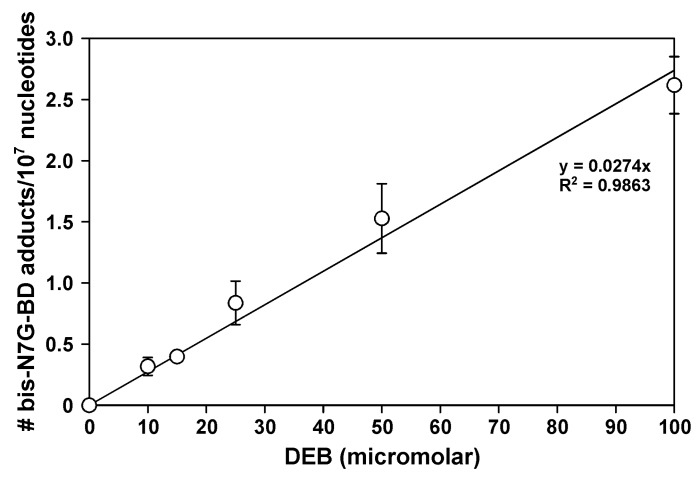
Concentration-dependent formation of *bis*-N7G-BD adducts in wild-type V79 cells treated with increasing amounts of DEB (10–100 µM). #, adduct numbers. Results represent average ± the standard error of the mean, *n* = 2.

DEB-induced ICL formation involves the N-7 residues on guanines on the opposite strands of 5′-GNC-3′ trinucleotides to form 1,4-*bis*-(guan-7-yl)-2,3-butanediol lesions (*bis*-N7G-BD, Scheme 1) [4]. We have previously shown that these lesions can be accurately quantified in cells and tissues by isotope dilution high pressure liquid chromatography electrospray ionization tandem mass spectrometry (HPLC-ESI^+^-MS/MS) following their release from the DNA backbone via neutral thermal hydrolysis [37,43]. In the present study, HPLC-ESI^+^-MS/MS assay was used to quantify time-dependent formation and removal of *bis*-N7G-BD in the cell lines described above. Our initial HPLC-ESI^+^-MS/MS analyses have revealed that *bis*-N7G-BD formation in V79 cells treated with increasing concentrations of DEB (0, 10, 15, 25, and 100 µM) was concentration-dependent, with adduct numbers ranging between 0 and 2.6 adducts per 10^7^ nucleotides (Figure 3). While this approach does not allow us to distinguish interstrand from intrastrand *bis*-N7G-BD crosslinks, our previous studies have shown that *SS*-, *RR*-DEB used in this work almost exclusively forms interstrand DNA–DNA cross-links [4,36].

**Figure 4:**
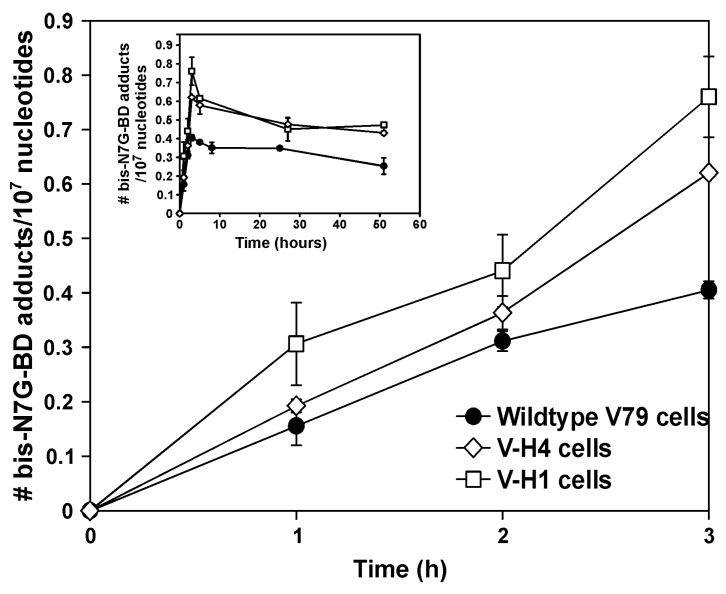
Time course for the formation (main panel) and repair (insert) of *bis*-N7G-BD cross-links in V79 (circles), V-H1 (squares), and V-H4 (diamonds) cells. Cells were exposed to 15 µM DEB for 1, 2, or 3 h. At the indicated times, chromosomal DNA was isolated, and *bis*-N7G-BD adduct levels were quantified by HPLC-ESI^+^-MS/MS as described in the Methods section. Results represent average ± the standard error of the mean, *n* = 2. Insert: ICL dynamics following DEB removal. Following treatment, DEB was removed and replaced with fresh growth media. DNA was isolated, and *bis*-N7G-BD adduct levels were determined as described above.

The pattern of altered sensitivity to DEB-induced cell death presented in Figure 1 led us to hypothesize that defects in repair of DEB-induced adducts in in V-H1 and V-H4 cells would result in increased levels of *bis*-N7G-BD crosslinks in these cells as compared to wild-type V79 cells. We further speculated that increases in cross-links would be particularly pronounced in the V-H4 clone. To test these predictions, we examined ICL formation and removal in V-H1, V-H4, and V79 clones treated with DEB. Cells were treated with 15 µM DEB for 3 h and then allowed to recover in DEB-free media for up to 48 h. Cells were harvested at various time points during and following exposure, and the amounts of *bis*-N7G-BD in chromosomal DNA were determined by isotope dilution HPLC-ESI^+^-MS/MS [43]. *Bis*-N7G-BD adduct numbers in cells exposed to DEB increased nearly linearly during the 3 h exposure period (Figure 4). At the end of this period, *bis*-N7G-BD adduct levels were the highest in the V-H1 cell line (0.76 ± 0.07 adducts/10^7^ nucleotides), were intermediate in V-H4 cells (0.62 ± 0.01 adducts/10^7^ nucleotides, and were the lowest in V79 cells (0.40 ± 0.01 adducts/10^7^ nucleotides) (Figure 4).

Analyses of the slope of linear regression curves revealed that ICLs formed in V79 cells with an apparent rate of 40 GG crosslinks per cell per hour (calculated from the data in Figure 4 based on an approximate haploid genome size of Chinese hamster cells of 2.3 gb). By comparison, the rates of ICL formation in V-H1 and V-H4 clones were ~70 and ~55 crosslinks/cell/h, respectively. Given that these cell lines are isogenic clones, the number of ICLs formed in the first 3 h most likely do not reflect the number of adducts induced, but rather lesions that were not repaired during drug treatment. Overall, our results reflect significantly increased levels of *bis*-N7G-BD adducts in the NER-deficient V-H1 cell lines compared to isogenic control at all three time points during drug treatment (T1, T2, T3 in Figure 4, *p* < 0.05) while FA-deficient VH-4 cells showed a significant increase in ICLs only at the time of drug removal (T3 in Figure 4, *p* < 0.05).

Upon DEB removal, *bis*-N7G-BD levels decreased slowly, with kinetics similar to the rates of spontaneous (i.e., non-enzymatic) depurination [37,43]. Substantial numbers of adducts (0.3–0.5 adducts/10^7^ nucleotides) were still present 48 h after DEB removal (Figure 4, insert), suggesting that a fraction of DEB-induced DNA–DNA cross-links persist in mammalian cells for extended periods of time. At all time points following removal of DEB, the levels of residual *bis*-N7G-BD in V-H1 cells were significantly higher than those detected in wild-type cells (Figure 4, insert, *p* < 0.05).

### 2.4. Bis-N7G-BD Adduct Formation in DEB-Treated Human Cells

The presence of elevated levels of ICLs in DEB-sensitive V-H4 hamster cells as compared to the isogenic, DEB-resistant V79 clone is consistent with the hypothesis that defects in adduct removal may underlie cellular sensitivity of the former cells to DEB. However, our observation that adduct levels did not significantly vary between the highly sensitive V-H4 cells as compared to the substantially less sensitive V-H1 clone appears to be inconsistent with this hypothesis. To shed further light on this question, we examined the kinetics of ICL formation and removal in human cells proficient or deficient in FA and NER genes.

This series of experiments was performed in human cell lines deficient in the *FANCD2* gene (PD20), the NER *XPA* gene (XPA), and on gene-corrected clones derived from these respective cell lines (see Materials and Methods). We first performed direct-cell counting cytotoxicity experiments that indicated both NER and FA-deficient human cell lines were significantly more sensitive to DEB than their corrected clones (Figure S1). *FANCD2*-deficient clones showed increased cellular sensitivity to DEB-induced cell death by nearly two-fold (LC_50_ 24 vs. 44 µM) while *XPA*-deficient clones were approximate 40% more sensitive (LC_50_ 40 vs. 55 µM) than their gene-corrected counterparts (Figure S1). While inactivation of FA and XP-genes in a human genetic background exerted a substantially smaller influence on DEB sensitivity than was observed in the hamster cells, the basic finding that FA-gene-deficient cells are more sensitive to DEB-induced cell death than NER-deficient clones was conserved in both species. We consequently performed an additional series of experiments to examine ICL formation and removal in wild-type and FA or XP-deficient human cell lines. These results revealed that the kinetics of ICL formation and removal in *FANCD2*- or *XPA*-deficient clones did not significantly differ from that observed in isogenic wild-type control lines (Figure S2). It is noteworthy that in hamster cells, inactivating mutations in NER and FA genes lead to elevated levels of ICL formation following DEB exposure, whereas in human cells inactivation of these genes have no effect on the kinetics of ICL formation or removal. Overall, the ICL kinetics data obtained from human and hamster DEB-treated cells are incompatible with the hypothesis that sensitivity to DEB is determined by the efficiency with which cells remove drug-induced ICLs.

### 2.5. Formation and Repair of DEB-Induced DNA Double Strand Breaks

**Figure 5:**
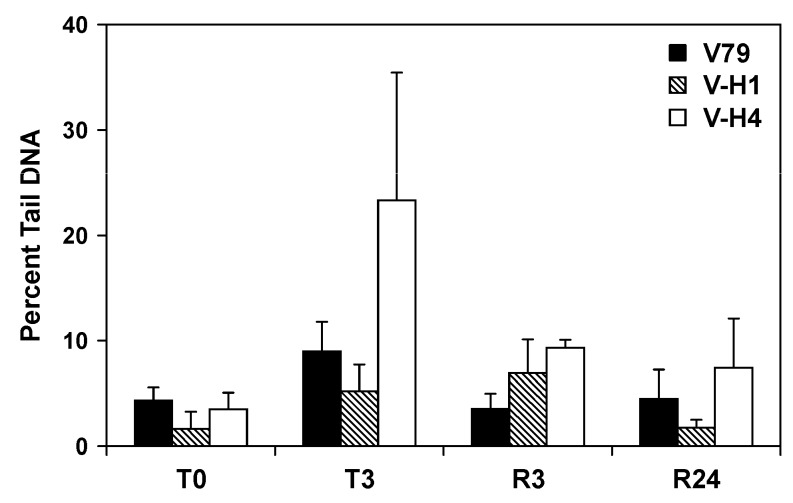
Formation and repair of DEB-induced DNA double strand breaks in V79, V-H1, and V-H4 cells. Single cell electrophoresis (comet assays) was performed on cells prior to and following exposure to 15 µM DEB as described in the Methods section. The data represent the percent of cellular DNA present in the “comet tail” plus the SEM, *n* = 3 or more. T0; pre-DEB exposure, T3; following 3 h of DEB exposure, R3; 3 h post-DEB exposure, R24; 24 h post-DEB exposure.

To gain further insight into the mechanism(s) underlying the differential sensitivity of V79, V-H1, and V-H4 fibroblasts toward DEB-mediated toxicity, we examined chromosomal DNA double strand break formation and rejoining in cells exposed to 15 µM DEB. First, single cell gel electrophoresis (“comet assays”) were performed on V79, V-H1, and V-H4 clones prior to treatment with DEB (T0), at the conclusion of the 3 h exposure (T3), and at 3 (R3) and 24 (R24) h post-removal of DEB. The results depicted in Figure 5 (black bars) show that immediately following exposure to DEB, V79 cells display a small increase in comet tail DNA, indicating the presence of slightly elevated levels of chromosomal DNA double strand breaks. This increase in comet tail DNA peaks shortly after the end of the 3 h DEB treatment and rapidly diminishes to pre-treatment levels. The comet tail analysis of DEB-treated V-H1 cells (striped bars) closely resembled that observed for V79 cells in terms of overall magnitude but displayed a slightly delayed kinetics as compared to V79 cells. In contrast, the levels of comet tail DNA in DEB-treated V-H4 cells were three-fold greater than those in similarly treated V79 cells. In V-H4 cells, the highest levels of DNA double-strand breaks were observed at the time point immediately after DEB removal (T3 in Figure 5, *p* < 0.05) and rapidly declined post-treatment (white bars in Figure 5). However, the numbers of DSBs in V-H4 cells remained elevated above pre-exposure levels for at least 24 h post-DEB treatment.

**Figure 6:**
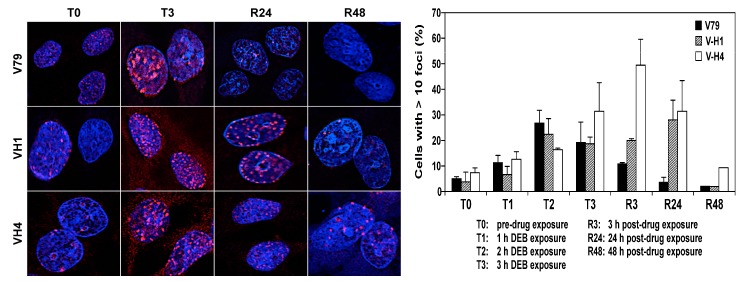
Images (**left**) and dynamics (**right**) of phosphorylated histone H2AX-containing foci in V79, V-H1 and V-H4 cells prior to and following exposure to 15 µM DEB. Cells were exposed to 15 µM DEB for 3 h and subsequently DEB was removed and replaced with fresh growth media. At the indicated times, immunocytochemistry was performed using a rabbit polyclonal antibody specific for phospho-H2AX, as described in the Methods section. Time points are the same as Figure 3. The data represent the percentage of cells within randomly selected microscope fields that contained 10 or more foci, plus the standard error of the mean, *n* = 3. * *p* < 0.05.

To confirm the relationship between ICL processing and chromosomal double strand break formation, we examined the kinetics of phospho-H2AX foci formation in V79, V-H1, and V-H4 cell lines during and after treatment with 15 µM DEB (Figure 6). Immunocytochemistry was performed using an antibody specific for phosphorylated histone H2AX (γ-H2AX), which is known to form foci at DNA double strand breaks [44]. The fraction of cells within randomly selected microscope fields that contained more than 10 γ-H2AX foci was calculated as described in the Materials and Methods. This analysis was performed prior to exposure (T0), at 1 (T1), 2 (T2), and 3 (T3) h of exposure to DEB, as well as 3 (R3) 24 (R24) and 48 (R48) h post-DEB exposure (see Figure 6 for representative images). An antibody specific for cyclin B1 was used to determine position within the cell cycle of DEB-treated V79 cells (2 h treatment) containing 10 or more foci. This analysis revealed that the majority of cells containing 10 or more foci were in the G2 (78%) or G1/S (14%) phases of the cell cycle, with the remaining 8% of cells in M phase. This is consistent with the data presented in Figure 2 that showed an increase of FA-deficient cells in G2/M cell cycle arrest, most likely due to the presence of ICL-induced double-strand breaks.

We found that γ-H2AX foci equally formed and disappeared rapidly in DEB-treated wild-type cells (V79) (black bars in Figure 6). The peak of focus formation occurred during the second hour of exposure, and by 24 h post-DEB treatment, foci levels had returned to basal levels. The kinetics of γ-H2AX foci (Figure 6) and comet tail DNA levels (Figure 5) were similar, consistent with the interpretation that both of these endpoints reflect chromosomal DNA double strand breaks. At initial time points, the dynamics of γ-H2AX foci formation in V-H1 cells (striped bars in Figure 6) were similar to those observed in V79 cells. However, significant differences were observed post-treatment. Unlike V79 cells, γ-H2AX foci in V-H1 cells persisted at elevated levels for at least 24 h (striped bars in Figure 6). The most substantial differences were observed for V-H4 cells, where the magnitude of DEB-induced γ-H2AX foci was significantly higher than for the other two clones (white bars in Figure 6, *p* < 0.05). These results are consistent with comet assay data (Figure 5) and are indicative of an increased persistence of DNA double strand breaks in V-H4 clones, which are deficient in the FA repair pathway.

### 2.6. Effect of a Non-Homologous End-Joining (NHEJ) Inhibitor on Cellular Sensitivity of V79 and V-H4 Cells to DEB

Two recent papers reported that inactivation of a number of NHEJ genes can rescue, or partially rescue, the hyper-sensitivity phenotype of FA-deficient clones to DNA cross-linking agents [45,46], suggesting that a function of the FA pathway is to prevent inappropriate end-joining repair of double strand breaks induced by cross-linking agents. To explore this possibility, we determined the influence of the DNA-PK inhibitor NU7026 on cellular survival of DEB-treated PD20 and their gene-corrected control. As the results in Table 1 reveal, exposure to 10 µM NU7026 had no significant effect on the sensitivity of either clone to DEB. To confirm this observation, we performed an additional series of experiments in which we examined the influence of NU7026 on hamster V79 (wild-type) and V-H4 (*FANCA*-deficient) clones. Control experiments confirmed that this concentration of NU7026 inhibits cellular DNA-PK activity in these cells [47] As was the case with human cells, inhibition of DNA-PK had no significant effect on the sensitivity of human wild-type or *FANCA*-deficient cells (Table 1).

## 3. Discussion

ICL repair is required for cell survival because of the ability of these toxic lesions to block DNA replication and transcription. Current models of ICL repair in mammalian cells support the roles of NER and FA repair pathways [29]. For example, cells derived from Chinese hamsters or humans that are deficient in the FA pathway are hypersensitive to the cytotoxic effects of a variety of DNA cross-linking agents [24,28]. Mammalian cell clones deficient in NER pathway members, especially *ERCC1* and *XPF*, show an enhanced sensitivity to ICL-forming agents such as cisplatin and cyclophosphamide [29]. Enoiu et al. [14] have shown that both transcription-coupled NER and FA recombinational repair pathways play a role in protecting cells from death induced by cisplatin. However, the relative contributions of the two repair pathways to ICL removal are still under debate.

Cellular sensitivity of DNA repair mutants towards ICL-inducing agents has often been used to judge the relative importance of various repair pathways to ICL removal [24]. However, it should be noted that in addition to ICL lesions, cross-linking agents such as cisplatin and nitrogen mustards induce large numbers of DNA monoadducts, which may be substrates for repair. In fact, DNA monoadducts represent 90–95% of DNA adducts formed upon treatment with *bis*-akylating agents, while ICL adducts account for 1–3% of the total [33,34]. Since DNA monoadducts and other types of DNA damage such as DNA-protein cross-links can contribute to the observed toxicity, the results of such cellular viability studies may be ambiguous and difficult to interpret.

Previous work has utilized mass spectrometry to quantify formation of ICL induced by psoralens and ultraviolet A (UVA) irradiation [48,49]. In the present study, we employed a mass spectrometry based methodology developed in our laboratory to accurately quantify the formation and repair of *bis*-N7G-BD cross-links in DEB-treated wild-type, NER-deficient, and FA-deficient clones. Due to the fact that the vast majority (~95%) of crosslinks produced by *d*,*l* DEB are of the interstrand type [37,43], this strategy permitted us to gain a new insight into the roles of NER and FA kinetics of ICL formation and removal. Combining this mass spectrometry methodology with parallel studies measuring cell survival, cell cycle dynamics, and the kinetics of DSB formation and rejoining has permitted us to establish a temporal relationship between ICL removal and DSB formation in the wild-type, NER-deficient, and FA-deficient clones.

We found that the numbers of ICL lesions increased in Chinese hamster-derived cells in a linear fashion during the 3 h exposure to DEB (Figure 4). Importantly, *bis*-N7G-BD cross-links accumulated at a faster rate in FA-deficient V-H4 cells and NER-deficient V-H1 cells as compared to the wild-type V79 clone (Figure 4). Since these clones are isogenic, differences in ICL accumulation reflect altered removal kinetics of chromosomal lesions that are formed at identical rates in the three cell lines. Notably, the vast majority of ICL removal occurred during the 3 h of treatment and in the first hours following DEB removal. The remaining lesions are fairly stable and are still observed after a 50 h incubation (Figure 4, insert). Similarly, Paz et al. noted little change in removal of ICLs induced by mitomycin C in EMT6 mouse mammary tumor cells 24 h post-incubation [50].

These results indicate that ICL removal is maximally effective in V79 wild-type cells, with slower rates of removal observed in both the V-H1 and V-H4 clones. Unexpectedly, ICL removal kinetics in these three cell lines (Figure 4) did not correlate with cell survival (Figure 1). The most DEB-sensitive clone (V-H4) accumulated ICLs at a faster rate than wild-type V79 clones and was characterized by substantially elevated levels of *bis*-N7G-BD throughout the duration of the 48 h post-exposure (Figure 4). However, V-H1 cells, which are only modestly more sensitive to DEB than the V79 cells (Figure 1), showed the highest levels of ICLs (Figure 4).

Experiments performed on human cell lines revealed that, unlike in hamster cells, inactivation of the NER (*XPA*) or FA (*FANCD2*) pathways had no significant effect on the kinetics of DEB-induced ICL formation or removal (Figure S2). DEB-induced ICL levels in *FANCD2*-deficient PD20 cells and their gene-corrected counterpart cells reached their highest levels at the end of the 3 h drug-treatment and then declined with linear kinetics over a subsequent three-day period. Parallel experiments performed in *XPA*-deficient and gene-corrected human cells revealed a similar pattern, with a slight, but not statistically significant, increase in ICL levels in *XPA*-deficient cells observed 3 h following removal of DEB. Again, as with the PD20 cells, ICL levels declined in both the NER-proficient and NER-deficient human with near-linear kinetics for the subsequent three-day period (Figure S2). Despite these species-specific differences in the kinetics of DEB-induced ICL formation and removal between wild type, NER-deficient, and FA-deficient clones, it is nevertheless clear that there is no obvious connection between drug sensitivity and the rate of ICL removal.

We, therefore, propose that the enhanced sensitivity to DEB-induced cell death observed in human and Chinese hamster cells deficient in the FA pathway is attributable to a defect in their ability to appropriately re-join ICL-induced DSBs. Consistent with this model, following exposure to DEB, chromosomal DSBs and γ-H2AX foci rapidly appear and disappear in the wild-type clone (V79), suggesting that DSBs formed during ICL removal are rapidly repaired. In contrast, DSBs are much more abundant and persist longer in FA-deficient V-H4 clones (Figure 5 and Figure 6). Given the hypersensitivity of FA cells to ICL-forming agents and their relative insensitivity to X-rays we propose that these clones suffer from a defect in re-joining DSB repair intermediates created following ICL removal.

Our observation of elevated levels of ICLs in DEB treated NER-deficient Chinese hamster cells (Figure 4), along with the slightly elevated ICLs observed in the human *XPA*-deficient clone (Figure S2) is consistent with the interpretation that NER plays a role in ICL repair [14]. Liu et al. also reported diminished repair of ICLs formed by 8-methoxypsoralen 24 h after treatment in *XPA*-deficient human skin fibroblasts [51]. However, the finding that these cells are only modestly more sensitive to DEB induced cell death than wild-type cells suggests that cells possess an NER-independent mechanism that is capable of removing these ICLs, albeit with diminished efficiency.

It has also been suggested that in the absence of functional FA DNA damage response, DSBs formed following ICL removal are directed to a “default” error-prone NHEJ pathway, which causes chromosomal rearrangements and cell death. Pace et al. have reported that inactivation of the NHEJ gene *Ku70* in *FANCC*-deficient chicken DT40 cells partially rescued the latter’s sensitivity to cisplatin-induced cell death [45]. Consistent with this finding, Adamo et al. [46] found that inactivation of the NHEJ gene *DNA ligase IV* in clones of *Caenorhabditis elegans* (*C. elegans*) deficient in the *FANCD2* gene restored wild-type sensitivity levels to cisplatin. These authors also observed that inhibition of the NHEJ factor DNA-dependent protein kinase (*DNA-PK*) rescued the mitomycin C hypersensitivity phenotype in human-derived HeLa or MO59K clones expressing *FANCD2* RNAi and partially rescued mitomycin C sensitivity in mouse cells null for either the *FANCA* or *FANCC* genes [46]. In contrast, murine cells lacking both *Ku80* and *FANCD2* genes exhibited greater sensitivity to mitomycin C and cisplatin than did clones deficient in either gene alone [52]. Similarly, Howard et al. reported that inactivation of the *Ku70* gene enhanced the cisplatin sensitivity of *FANCA*-deficient murine cells [53]. In a study by Pace et al. [45], inactivation of either the *DNA ligase IV* or *DNA PK* genes influenced the cisplatin-sensitivity phenotype of *FANCC* deficient in *C. elegans*. In the present work, *DNA PK* inhibition had no effect on toxicity of DEB in either FA-proficient or FA-deficient mammalian cells (Table 1). Taken together, these results lead us to conclude that a complex interaction of both the HR and end-joining repair pathways ultimately determines the fate of DSBs produced during xenobiotic-induced ICL repair.

## 4. Materials and Methods

DEB is a known human and animal carcinogen and should be handled with adequate safety precautions in a well-ventilated fume hood strictly following its material safety data sheet.

LC-MS grade acetonitrile, methanol, and water were obtained from Fisher Scientific (Pittsburgh, PA, USA). NU7026 was from Selleckchem (Houston, TX, USA). All other chemicals and solvents were obtained from Sigma-Aldrich (St. Louis, MO, USA). Anti-H2AX antibody was obtained from Bethyl Laboratories (Montgomery, TX, USA), anti-cyclin B1 antibody was from Santa Cruz Biotechnology (Dallas, TX, USA). Goat anti-mouse secondary antibody (alexa fluor 488 conjugated) was from Thermo Scientific (Waltham, MA, USA), goat anti-rabbit secondary antibody (alexa fluor 633 conjugated) was from Invitrogen (Grand Island, NY, USA). *Bis*-N7G-BD and ^15^N_10_-*bis*-N7G-BD (internal standard for mass spectrometry) were prepared in our laboratory as described previously [36,37,43], and their concentrations were determined by UV spectrophotometry (ε_252_ = 15700, pH 1). 

### 4.1. Cell Culture

Chinese hamster lung fibroblast cell lines V79 (GM16136), V-H4 (GM16142), and V-H1 (GM16141) were obtained from the Coriell Institute for Medical Research (Camden, NJ, USA). V79 are wild-type cells from which the V-H4 and V-H1 clones were derived following ethylnitrosourea-induced mutagenesis [39,54,55]. V-H4 cells are deficient in the hamster homolog of the human Fanconi anemia complementation group A (*FANCA*) gene [56,57]. V-H1 cells belong to complementation group 2 of nucleotide excision repair-deficient mutants [58]. These cells are deficient in the *ERCC2* gene, which codes for the *XPD* protein involved in the helicase activity that unwinds duplex DNA during nucleotide excision repair [57]. Cells were grown to 80–90% confluence on tissue culture dishes in Ham’s F-12 modified essential Eagle’s media (Life Technologies, Grand Island, NY, USA) supplemented with 9% fetal bovine serum. A human dermal skin-derived clone [59] obtained from a patient with biallelic mutations in the *FANCD2* gene (PD20), and a retrovirally-corrected clone, PDcorr [60] were kind gifts from Dr. Alexandra Sobeck, University of Minnesota. Immortalized human dermal fibroblasts from an XP patient with inactivating mutations in the NER *XPA* gene, as well as from a gene-corrected clone derived from this line, were obtained from the Coriell institute (GM04312). Human cells were cultured in Dulbecco’s modified Eagle’s media (Life Technologies) supplemented with 9% fetal bovine serum. All cells were maintained in a humidified atmosphere of 5% carbon dioxide, 95% air, at 37 °C.

### 4.2. Cytotoxicity Assays

For direct-counting assays, cells (V79, V-H1 and V-H4) were seeded into 6 cm dishes at a density of 0.5 × 10^6^ cells/dish. On the following day, cells were exposed to various concentrations of DEB (1–45 µM) for 3 h in serum-free media. Following treatment, the media was replaced with DEB-free normal growth media. Twenty-four hours later, cells were harvested and counted using a hemocytometer. Trypan blue exclusion confirmed that ≥99% of recovered cells were viable. Percent cell survival was calculated by dividing the number of cells obtained from DEB-treated cultures compared to the corresponding number of cells recovered from control cultures not exposed to DEB. All experiments were performed in triplicate, and the results represent the average ± the standard error of the mean from three or more independent experiments. Direct-counting assays were also performed on human XPA and PD20 cell lines as well as their isogenic controls. These experiments were conducted as described above using 1–30 µM DEB.

For clonogenic assays, V79, V-H1, and V-H4 cells were plated in triplicate in 6 cm dishes at a density of 500 cells/dish. On the following day, the cells were exposed to various concentrations of DEB (1–45 µM) for 3 h in serum-free media and then replaced with DEB-free normal growth media. Following 7 days of growth, the colonies were fixed and stained with 1% crystal violet in 95% ethanol. Colonies (>50 cells) were counted manually, and percent colony formation was calculated by dividing the number of colonies obtained from DEB-treated cultures compared to the corresponding number of colonies recovered from control cultures not exposed to DEB. Experiments were performed in triplicate, and the results represent the average ± the standard error of the mean from 3 independent experiments.

To examine the influence of inactivating cellular non-homologous DNA end-joining activity, cytotoxicity experiments were performed in the presence or absence of the DNA-PK inhibitor NU7026 (Selleckchem, Houston, TX, USA). Human PD20 and PDcorr cells and hamster V79 and V-H4 cells were exposed to DEB (5 or 15 µM, respectively, for human or hamster cells) for 1 h in the presence or absence of 10 µM NU7026, in triplicate. At the end of the 1 h incubation, drug-containing media was replaced with drug-free media, allowed to recover for 24 h, and then counted as described above. The results from these experiments represent an average ± the standard error of the mean from two independent experiments.

### 4.3. Effects of DEB Exposure on Cell Cycle Distribution

V79, V-H4, and V-H1 cells were plated at a density of 0.5 × 10^6^ cells/dish on 10 cm dishes in normal growth media. On the following day, media was removed and replaced with fresh media (control) or with media containing 15 μM DEB. Following a 3 h incubation at 37 °C, dishes were replaced with fresh media and allowed to recover for 24 h. Cells were then washed in phosphate buffered saline (PBS) and fixed in 70% ice-cold ethanol. After fixation, cells (1 × 10^6^ per mL) were resuspended in PBS containing 100 μg/mL RNAse A and stained with 40 μg/mL propidium iodide. Flow cytometry was performed using a BD LSR II/Fortessa H4710 instrument, with analysis performed using FlowJo data analysis software (V10 for Mac, FlowJo LLC, Ashland, OR, USA).

### 4.4. Bis-N7G-BD Adduct Detection in DEB-Treated Cells

V79 cells (6 million cells, in duplicate) were treated with 0, 10, 15, 25, 50 or 100 µM DEB for 3 h. DEB-containing media was removed, and the cells were washed with PBS. Cells were harvested with PBS, sedimented, and stored at −20 °C until DNA extraction and adduct analysis. V79, V-H4, and V-H1 hamster cells (6–8 million, in duplicate) were treated with 15 µM DEB for 0, 1, 2 and 3 h. Following treatment, DEB-containing media was replaced with fresh media, and the cells were allowed to recover for 2, 24, or 48 h to allow for adduct repair. Human XPA, XPAcorr, PD20, and PDcorr cell lines were treated with 15 µM DEB for 3 h and allowed to recover for 0, 3, 24, or 72 h. To quantify the *bis-*N7G-BD adducts remaining at these time points, cells were harvested with 5 mL of PBS, sedimented, and stored at −20 °C until DNA extraction and adduct analysis as described below.

DNA extraction from cells was performed using standard phenol-chloroform extraction as described previously [61]. DNA concentrations were determined by UV spectrophotometry (Thermo Scientific) based on the absorbance at 260 nm. DNA purity was assessed from A_260_/A_280_ absorbance ratios, which were typically between 1.8 and 1.9. DNA (50–150 µg) was re-suspended in water (200 µL) and spiked with ^15^N_10_-*bis*-N7G-BD (50 fmol, internal standard for mass spectrometry). Samples were subjected to neutral thermal hydrolysis (70 °C for 1 h) to release *bis*-N7G-BD adducts from the DNA backbone as free base conjugates. Following ultrafiltration to remove partially depurinated DNA, *bis*-N7G-BD and ^15^N_10_-*bis*-N7G-BD were isolated by offline HPLC as described elsewhere [61]. HPLC fractions corresponding to *bis*-N7G-BD and its internal standard were dried under reduced pressure, reconstituted in water (25 µL), and subjected to nanoLC-ESI^+^-MS/MS analysis as described previously [62]. Selected reaction monitoring transitions used for quantitation of *bis*-N7G-BD were *m*/*z* 389.1 [M + H]^+^ → 238.1 [M + H − Gua]^+^, *m*/*z* 152.1 [Gua + H]^+^ for *bis*-N7G-BD and *m*/*z* 399.1 [15N10-M + H]^+^ → 243.1 [M + H − [15N5]Gua]^+^, *m*/*z* 157.1 [15N5-Gua + H]^+^ for ^15^N_10_-*bis*-N7G-BD. Quantitation was conducted using nanoLC-ESI^+^-MS/MS areas in extracted ion chromatograms corresponding to the analyte and its internal standard [61]. Calibration curves were constructed with authentic standards of *bis*-N7G-BD and ^15^N_10_-*bis*-N7G-BD.

### 4.5. Native Comet Assay

Chromosomal DNA strand breaks were detected by performing comet assays [63] under neutral conditions using the CometAssay kit from Trevigen (Gaithersburg, MD, USA). Hamster cells were incubated in the presence or in the absence of 15 µM DEB in serum-free media for 3 h at 37 °C. Culture media was removed, and the cells were incubated at 37 °C in DEB-free growth media for times the indicated (0, 3, and 24 h). Cells were harvested and suspended in PBS at a density of ~1 × 10^6^ cells/mL. Briefly, 10 µL of cell suspension was mixed with 90 µL of 0.5% low melting temperature agarose (GIBCOBRL, Grand Island, NY, USA) in PBS and layered on top of microscope slides pre-coated with 1% normal agarose. Slides were placed flat at 4 °C in the dark for 15 min and then lysed overnight at 4 °C in the dark in lysis buffer (10 mM Tris, pH 10, 0.1 M ethylenediaminetetraaceticacid (EDTA), 2.5 M NaCl, 1% *N*-laurylsarcosine, 1% Triton-X 100). Following electrophoresis in TRIS Borate EDTA buffer (8.9 mM TRIS base, 89 mM boric acid, and 2.5 mM EDTA) for 30 min at 1 V/cm, slides were washed in DNA precipitation solution (1 M ammonium acetate in 95% ethanol) for 30 min at room temperature. Slides were then immersed in 70% ethanol for 30 min at room temperature, dried at 37 °C for 15 min, and then stained with 100 µL of diluted SYBR Green (1:10,000 in TE Buffer) for 30 min in the dark. Cells were imaged using a Zeiss Axioplan 2 upright microscope (ZEISS, Oberkochen, Germany). At least 50 cells were randomly selected from each treatment and blindly scored into categories 0 through 4 [64] based upon increasing fluorescence intensity of the comet tail relative to the head.

### 4.6. Immunocytochemistry for Visualization of γH2AX Foci and Cyclin B1

Immunocytochemistry was performed as described elsewhere [5,44] with some modifications. For all cell lines, 1 × 10^5^ cells were seeded in 3.5 cm dishes that contained sterilized glass cover slips (22 × 22 mm, No.1 Thickness, Thermo Scientific) and incubated overnight at 37 °C in normal growth media. On the following day, individual dishes were treated with serum-free medium in the presence or in the absence of 15 μM DEB in for 3 h at 37 °C. DEB-containing media was replaced with normal growth media, and the cells were maintained at 37 °C for recovery at various times (0, 3, 24, and 48 h). Following the removal of the media, ice cold 50% methanol: 50% acetone was added, and the cells were incubated for 8 min at 4 °C. Cells were then washed with ice-cold PBS three times and permeabilized with 0.1% Triton X-100 in PBS for 5 min at room temperature, followed by incubation in blocking buffer (0.1% Triton X-100, 0.2% skimmed dry milk in PBS) at 4 °C overnight in a humidified chamber. On the following day, samples were incubated with rabbit polyclonal antibody specific for phosphorylated H2AX (γH2AX, Bethyl Scientific, Montgomery, TX) at 1:10,000 dilution in blocking buffer for 1 h at room temperature. After washing three times with 0.1% Triton X-100 in PBS, cells were incubated with Alexa Fluor 633 conjugated goat anti-rabbit secondary antibody (Invitrogen) at a dilution of 1:2000 in blocking buffer for 1 h at 4 °C in the dark. Cells were washed with PBS and counterstained with 4′,6-diamidino-2-phenylindole dihydrochloride dihydrate, (DAPI, obtained from Sigma) at 1:10,000 dilution in PBS for 3 min. Stained cells were washed with ice-cold PBS three times. The slides were mounted with Fluoromount-G9R^®^ (SouthernBiotech, Birmingham, AL, USA), and the cover slip edges were sealed with clear nail polish. Cell cycle position of drug-treated cells was visualized by immunocytochemistry using a cyclin B1-specific antibody (Santa Cruz Biotechnology, diluted 1:500) followed by exposure to a goat anti-mouse antibody (ThermoScientific), essentially as described above.

Images were visualized with an Olympus FluoView FV1000 BX2 upright confocal microscope (60× oil immersion objective; Olympus, Tokyo, Japan) equipped with 633 nm HeNe excitation laser. Foci were counted in at least 50 cells per time point, and results from three independent experiments were averaged. Cells were scored into two categories, i.e., ≤10 and >10 foci/cell. The γ-H2AX foci were scored manually on coded samples in a blind manner. Data were expressed as the mean percentage of γ-H2AX-positive cells (±the standard error of the mean).

## 5. Conclusions

The current study suggests that that the increased sensitivity of FA-deficient hamster cells towards DNA cross-linking agents such as DEB may be due to the accumulation of DNA double strand breaks, which are created upon replication of ICL containing DNA. Additionally, the NER pathway contributes to ICL recognition and removal in mammalian cells, although NER deficiency has a smaller effect on cell viability in the presence of cross-linking agents.

## Figures and Tables

**Table 1:** Effect of DNA-PK inhibitor Nu7026 on 1,2,3,4-diepoxybutane (DEB)-induced cell death in human and hamster cells. Cells were exposed to DEB in the presence or absence of 10 µM Nu7026 and the percent cell death determined as described in the Methods section.

Cell Line	PDcorr (WT)		PD20 (-FANCD2)		V79 (WT)		V-H4 (-FANCA)	
Nu7026	-	+	-	+	-	+	-	+
Percent cell death (± SEM)	21 (± 1.4)	20 (± 7.8)	40 (± 2.8)	42 (± 8)	23 (± 1.4)	22 (± 1.4)	42 (± 2.1)	42 (± 3.5)

## Supplementary Materials

Supplementary materials can be found at www.mdpi.com/1422-0067/18/5/1086/s1.

## Abbreviations

ICL	Interstrand DNA–DNA cross-links
DSB	DNA double strand break
*bis*-N7G-BD	DNA double strand break
DEB	1,2,3,4-diepoxybutane
NER	Nucleotide excision repair
HR	Homologous recombinational repair
FA	Fanconi anemia
NHEJ	Non-homologous DNA end-joining
DNA-PK	DNA-dependent protein kinase
PBS	Phosphate buffered saline
DAPI	4′,6-diamidino-2phenylindole dihydrochloride dihydrate

## References

[1] Deans A.J., West S.C. (2011). DNA interstrand crosslink repair and cancer. Nat. Rev. Cancer.

[2] Kozekov I.D., Nechev L.V., Moseley M.S., Harris C.M., Rizzo C.J., Stone M.P., Harris T.M. (2003). DNA interchain cross-links formed by acrolein and crotonaldehyde. J. Am. Chem. Soc..

[3] Stone M.P., Cho Y.J., Huang H., Kim H.Y., Kozekov I.D., Kozekova A., Wang H., Minko I.G., Lloyd R.S., Harris T.M. (2008). Interstrand DNA cross-links induced by alpha, beta-unsaturated aldehydes derived from lipid peroxidation and environmental sources. Acc. Chem Res..

[4] Park S., Tretyakova N. (2004). Structural characterization of the major DNA-DNA cross-link of 1,2,3,4-diepoxybutane. Chem. Res. Toxicol..

[5] Vare D., Groth P., Carlsson R., Johansson F., Erixon K., Jenssen D. (2012). DNA interstrand crosslinks induce a potent replication block followed by formation and repair of double strand breaks in intact mammalian cells. DNA Repair.

[6] Eastman A. (1986). Reevaluation of interaction of *cis*-dichloro(ethylenediamine)platinum(II) with DNA. Biochemistry.

[7] Gargiulo D., Kumar G.S., Musser S.S., Tomasz M. (1995). Structural and function modification of DNA by mitomycin C. Mechanism of the DNA sequence specificity of mitomycins. Nucl. Acids Symp. Ser..

[8] Vare D., Johansson F., Persson J.O., Erixon K., Jenssen D. (2014). Quantification and repair of psoralen-induced interstrand crosslinks in human cells. Toxicol. Lett..

[9] Fleer R., Brendel M. (1982). Toxicity, interstrand cross-links and DNA fragmentation induced by “activated” cyclophosphamide in yeast: Comparative studies on 4-hydroperoxy-cyclophosphamide, its monofunctional analogon, acrolein, phosphoramide mustard, and nor-nitrogen mustard. Chem. Boil. Interact..

[10] Cole R.S. (1973). Repair of DNA containing interstrand crosslinks in *Escherichia coli*: Sequential excision and recombination. Proc. Natl. Acad. Sci. USA.

[11] Dronkert M.L., Kanaar R. (2001). Repair of DNA interstrand cross-links. Mutat. Res..

[12] Lehoczky P., McHugh P.J., Chovanec M. (2007). DNA interstrand cross-link repair in Saccharomyces cerevisiae. FEMS Microb. Rev..

[13] Muniandy P.A., Thapa D., Thazhathveetil A.K., Liu S.T., Seidman M.M. (2009). Repair of laser-localized DNA interstrand cross-links in G1 phase mammalian cells. J. Biol. Chem..

[14] Enoiu M., Jiricny J., Scharer O.D. (2012). Repair of cisplatin-induced DNA interstrand crosslinks by a replication-independent pathway involving transcription-coupled repair and translesion synthesis. Nucleic Acids Res..

[15] Dardalhon M., Averbeck D. (1995). Pulsed-field gel electrophoresis analysis of the repair of psoralen plus UVA induced DNA photoadducts in Saccharomyces cerevisiae. Mutat. Res..

[16] Magana-Schwencke N., Henriques J.A., Chanet R., Moustacchi E. (1982). The fate of 8-methoxypsoralen photoinduced crosslinks in nuclear and mitochondrial yeast DNA: Comparison of wild-type and repair-deficient strains. Proc. Natl. Acad. Sci. USA.

[17] Saffran W.A., Ahmed S., Bellevue S., Pereira G., Patrick T., Sanchez W., Thomas S., Alberti M., Hearst J.E. (2004). DNA repair defects channel interstrand DNA cross-links into alternate recombinational and error-prone repair pathways. J. Biol. Chem..

[18] De Silva I.U., McHugh P.J., Clingen P.H., Hartley J.A. (2000). Defining the roles of nucleotide excision repair and recombination in the repair of DNA interstrand cross-links in mammalian cells. Mol. Cell. Biol..

[19] Niedernhofer L.J., Odijk H., Budzowska M., van Drunen E., Maas A., Theil A.F., de Wit J., Jaspers N.G., Beverloo H.B., Hoeijmakers J.H.J. (2004). The structure-specific endonuclease Ercc1-Xpf is required to resolve DNA interstrand cross-link-induced double-strand breaks. Mol. Cell. Biol..

[20] Rothfuss A., Grompe M. (2004). Repair kinetics of genomic interstrand DNA cross-links: Evidence for DNA double-strand break-dependent activation of the Fanconi anemia/BRCA pathway. Mol. Cell. Biol..

[21] McHugh P.J., Sones W.R., Hartley J.A. (2000). Repair of intermediate structures produced at DNA interstrand cross-links in Saccharomyces cerevisiae. Mol. Cell. Biol..

[22] Zhang J., Walter J.C. (2014). , Mechanism and regulation of incisions during DNA interstrand cross-link repair. DNA Repair.

[23] Ho T.V., Scharer O.D. (2010). Translesion DNA synthesis polymerases in DNA interstrand crosslink repair. Environ. Mol. Mutagen..

[24] Walden H., Deans A.J. (2014). The Fanconi anemia DNA repair pathway: Structural and functional insights into a complex disorder. Annu. Rev. Biophys..

[25] Bogliolo M., Schuster B., Stoepker C., Derkunt B., Su Y., Raams A., Trujillo J.P., Minguillon J., Ramirez M.J., Pujol R. (2013). Mutations in *ERCC4*, encoding the DNA-repair endonuclease XPF, cause Fanconi anemia. Am. J. hum. Genet..

[26] Kashiyama K., Nakazawa Y., Pilz D.T., Guo C., Shimada M., Sasaki K., Fawcett H., Wing J.F., Lewin S.O., Carr L. (2013). Malfunction of nuclease ERCC1-XPF results in diverse clinical manifestations and causes Cockayne syndrome, xeroderma pigmentosum, and Fanconi anemia. Am. J. Hum. Genet..

[27] Scharer O.D. (2013). Nucleotide excision repair in eukaryotes. Cold Spring Harb. Perspect. Boil..

[28] Hoy C.A., Thompson L.H., Mooney C.L., Salazar E.P. (1985). Defective DNA cross-link removal in Chinese hamster cell mutants hypersensitive to bifunctional alkylating agents. Cancer Res..

[29] Wood R.D. (2010). Mammalian nucleotide excision repair proteins and interstrand crosslink repair. Environ. Mol. Mutagen..

[30] Mouw K.W., D’Andrea A.D. (2014). Crosstalk between the nucleotide excision repair and Fanconi anemia/BRCA pathways. DNA Repair.

[31] Crossan G.P., Patel K.J. (2012). The Fanconi anaemia pathway orchestrates incisions at sites of crosslinked DNA. J. Pathol..

[32] Cochrane J.E., Skopek T.R. (1994). Mutagenicity of butadiene and its epoxide metabolites: II. Mutational spectra of butadiene, 1,2-epoxybutene and diepoxybutane at the hprt locus in splenic T cells from exposed B6C3F1 mice. Carcinogenesis.

[33] Tretyakova N., Chiang S.Y., Walker V.E., Swenberg J.A. (1998). Quantitative analysis of 1,3-butadiene-induced DNA adducts in vivo and in vitro using liquid chromatography electrospray ionization tandem mass spectrometry. J. Mass Spectrum. JMS.

[34] Tretyakova N., Lin Y., Sangaiah R., Upton P.B., Swenberg J.A. (1997). Identification and quantitation of DNA adducts from calf thymus DNA exposed to 3,4-epoxy-1-butene. Carcinogenesis.

[35] Lawley P.D., Brookes P. (1967). Interstrand cross-linking of DNA by difunctional alkylating agents. J. Mol. Boil..

[36] Park S., Anderson C., Loeber R., Seetharaman M., Jones R., Tretyakova N. (2005). Interstrand and intrastrand DNA–DNA cross-linking by 1,2,3,4-diepoxybutane: Role of stereochemistry. J. Am. Chem. Soc..

[37] Goggin M., Sangaraju D., Walker V.E., Wickliffe J., Swenberg J.A., Tretyakova N. (2011). Persistence and repair of bifunctional DNA adducts in tissues of laboratory animals exposed to 1,3-butadiene by inhalation. Chem. Res. Toxicol..

[38] Franken N.A., Rodermond H.M., Stap J., Haveman J., van Bree C. (2006). Clonogenic assay of cells in vitro. Nat. Protoc..

[39] Zdzienicka M.Z., Arwert F., Neuteboom I., Rooimans M., Simons J.W. (1990). The Chinese hamster V79 cell mutant V-H4 is phenotypically like Fanconi anemia cells. Somat. Cell Mol. Genet..

[40] Kang S.G., Chung H., Yoo Y.D., Lee J.G., Choi Y.I., Yu Y.S. (2001). Mechanism of growth inhibitory effect of Mitomycin-C on cultured human retinal pigment epithelial cells: Apoptosis and cell cycle arrest. Curr. Eye Res..

[41] Seyschab H., Friedl R., Sun Y., Schindler D., Hoehn H., Hentze S., Schroeder-Kurth T. (1995). Comparative evaluation of diepoxybutane sensitivity and cell cycle blockage in the diagnosis of Fanconi anemia. Blood.

[42] Nieto A., Cabrera C.M., Catalina P., Cobo F., Barnie A., Cortes J.L., Barroso del Jesus A., Montes R., Concha A. (2007). Effect of mitomycin-C on human foreskin fibroblasts used as feeders in human embryonic stem cells: Immunocytochemistry MIB1 score and DNA ploidy and apoptosis evaluated by flow cytometry. Cell Biol. Int..

[43] Goggin M., Loeber R., Park S., Walker V., Wickliffe J., Tretyakova N. (2007). HPLC-ESI^+^-MS/MS analysis of N7-guanine-N7-guanine DNA cross-links in tissues of mice exposed to 1,3-butadiene. Chem. Res. Toxicol..

[44] Clingen P.H., Wu J.Y., Miller J., Mistry N., Chin F., Wynne P., Prise K.M., Hartley J.A. (2008). Histone H2AX phosphorylation as a molecular pharmacological marker for DNA interstrand crosslink cancer chemotherapy. Biochem. Pharmacol..

[45] Pace P., Mosedale G., Hodskinson M.R., Rosado I.V., Sivasubramaniam M., Patel K.J. (2010). Ku70 corrupts DNA repair in the absence of the Fanconi anemia pathway. Science.

[46] Adamo A., Collis S.J., Adelman C.A., Silva N., Horejsi Z., Ward J.D., Martinez-Perez E., Boulton S.J., La Volpe A. (2010). Preventing nonhomologous end joining suppresses DNA repair defects of Fanconi anemia. Mol. Cell.

[47] Chesner L.N. (2017). University of Minnesota, Minneapolis, MN, USA.

[48] Lai C., Cao H., Hearst J.E., Corash L., Luo H., Wang Y. (2008). Quantitative analysis of DNA interstrand cross-links and monoadducts formed in human cells induced by psoralens and UVA irradiation. Anal. Chem..

[49] Cao H., Hearst J.E., Corash L., Wang Y. (2008). LC-MS/MS for the detection of DNA interstrand cross-links formed by 8-methoxypsoralen and UVA irradiation in human cells. Anal. Chem..

[50] Paz M.M., Ladwa S., Champeil E., Liu Y., Rockwell S., Boamah E.K., Bargonetti J., Callahan J., Roach J., Tomasz M. (2008). Mapping DNA adducts of mitomycin C and decarbamoyl mitomycin C in cell lines using liquid chromatography/ electrospray tandem mass spectrometry. Chem. Res. Toxicol..

[51] Liu S., Wang Y. (2013). A quantitative mass spectrometry-based approach for assessing the repair of 8-methoxypsoralen-induced DNA interstrand cross-links and monoadducts in mammalian cells. Anal. Chem..

[52] Bunting S.F., Callen E., Kozak M.L., Kim J.M., Wong N., Lopez-Contreras A.J., Ludwig T., Baer R., Faryabi R.B., Malhowski A. (2012). BRCA1 functions independently of homologous recombination in DNA interstrand crosslink repair. Mol. Cell.

[53] Howard S.M., Yanez D.A., Stark J.M. (2015). DNA damage response factors from diverse pathways, including DNA crosslink repair, mediate alternative end joining. PLoS Genet..

[54] Larminat F., Germanier M., Papouli E., Defais M. (2004). Impairment of homologous recombination control in a Fanconi anemia-like Chinese hamster cell mutant. Biol. Cell.

[55] Studzian K., Telleman P., van der Schans G.P., Zdzienicka M.Z. (1994). Mutagenic response and repair of cis-DDP-induced DNA cross-links in the Chinese hamster V79 cell mutant V-H4 which is homologous to Fanconi anemia (group A). Mutat. Res..

[56] Telleman P., Overkamp W.J., van Wessel N., Studzian K., Wetselaar L., Natarajan A.T., Zdzienicka M.Z. (1995). A new complementation group of mitomycin C-hypersensitive Chinese hamster cell mutants that closely resembles the phenotype of fanconi anemia cells. Cancer Res..

[57] Arwert F., Rooimans M.A., Westerveld A., Simons J.W., Zdzienicka M.Z. (1991). The Chinese hamster cell mutant V-H4 is homologous to Fanconi anemia (complementation group A). Cytogenet. Cell Genet..

[58] Kadkhodayan S., Salazar E.P., Ramsey M.J., Takayama K., Zdzienicka M.Z., Tucker J.D., Weber C.A. (1997). Molecular analysis of *ERCC2* mutations in the repair deficient hamster mutants UVL-1 and V-H1. Mutat. Res..

[59] Jakobs P.M., Fiddler-Odell E., Reifsteck C., Olson S., Moses R.E., Grompe M. (1997). Complementation group assignments in Fanconi anemia fibroblast cell lines from North America. Somat. Cell Mol. Genet..

[60] Timmers C., Taniguchi T., Hejna J., Reifsteck C., Lucas L., Bruun D., Thayer M., Cox B., Olson S., D’Andrea A.D. (2001). Positional cloning of a novel Fanconi anemia gene, *FANCD2*. Mol. Cell.

[61] Michaelson-Richie E.D., Ming X., Codreanu S.G., Loeber R.L., Liebler D.C., Campbell C., Tretyakova N.Y. (2011). Mechlorethamine-induced DNA-protein cross-linking in human fibrosarcoma (HT1080) cells. J. Proteome Res..

[62] Sangaraju D., Goggin M., Walker V., Swenberg J., Tretyakova N. (2012). NanoHPLC-nanoESI^+^-MS/MS quantitation of *bis*-N7-guanine DNA-DNA cross-links in tissues of B6C3F1 mice exposed to subppm levels of 1,3-butadiene. Anal. Chem..

[63] Fairbairn D.W., Olive P.L., O’Neill K.L. (1995). The comet assay: A comprehensive review. Mutat. Res..

[64] Kumaravel T.S., Vilhar B., Faux S.P., Jha A.N. (2009). Comet Assay measurements: A perspective. Cell Boil. Toxicol..

