# Supplementary material for: Cellular Repair of DNA–DNA Cross-Links Induced by 1,2,3,4-Diepoxybutane
Source: Int J Mol Sci. 2017 May 18;18(5):1086. doi: 10.3390/ijms18051086 (PMC5454995; doi:10.3390/ijms18051086)
Supplement: Supplementary file 1 [file ijms-18-01086-s001.pdf]

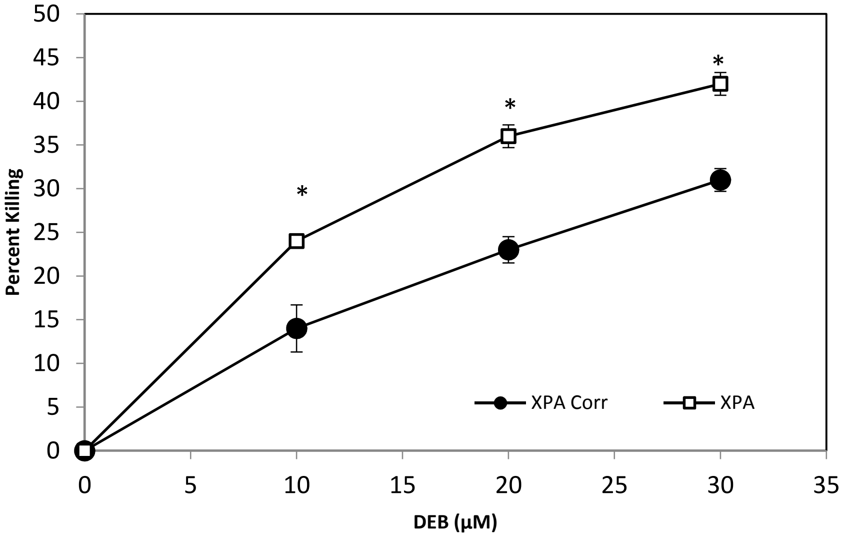
50
45
40
35
30
25
20
15
10
5
0
Percent Killing
0
5
10
15
20
25
30
35
DEB (µM)
XPA Corr
XPA
*
*
*

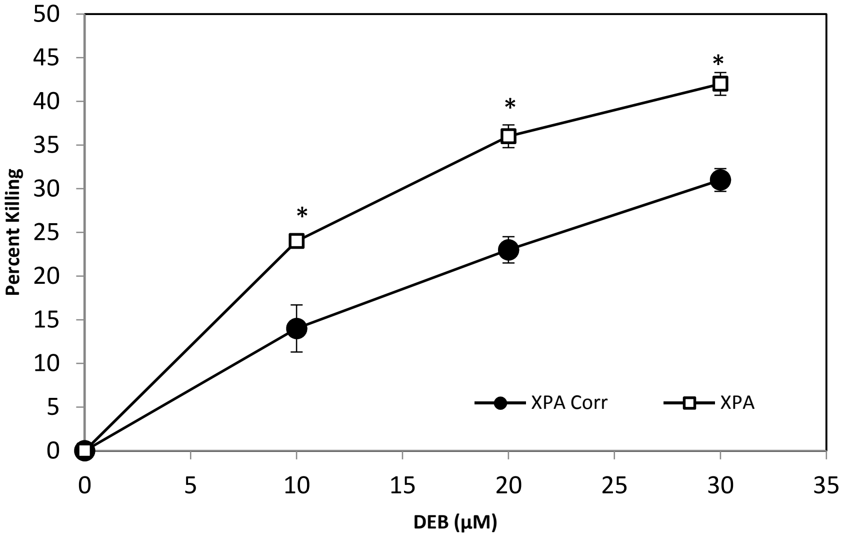
50
45
40
35
30
25
20
15
10
5
0
Percent Killing
0
5
10
15
20
25
30
35
DEB (µM)
XPA Corr
XPA
*
*
*

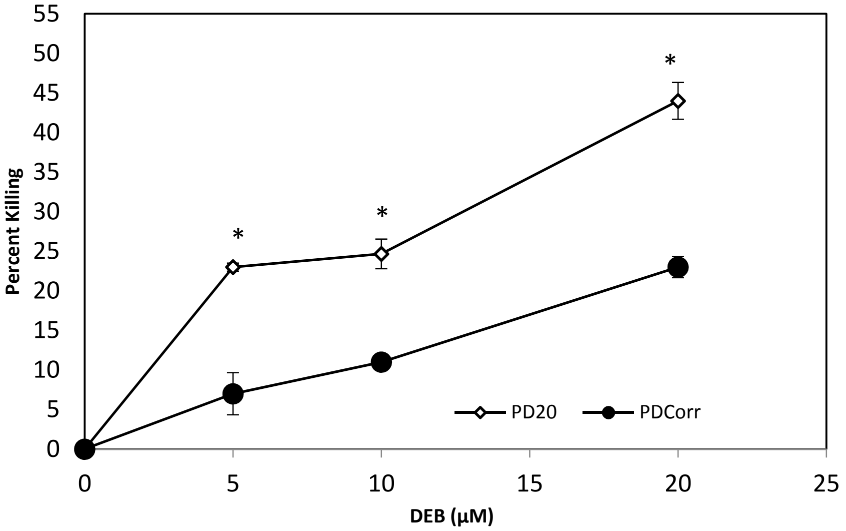
55
50
45
40
35
30
25
20
15
10
5
0
Percent Killing
0
5
10
15
20
25
DEB (µM)
PD20
PDCorr
*
*
*

Figure S1. Effects of exposure to DEB on cell death of human wild-type (circles), XPA (squares) and PD20 (diamonds) cells. The influence of exposure to various concentrations of DEB for 3 h on cell survival was determined through direct cell counting of trypan blue-excluding cells as described in the Methods section. Results represent average ± the standard error of the mean, N= 3 or more *P<0.05.

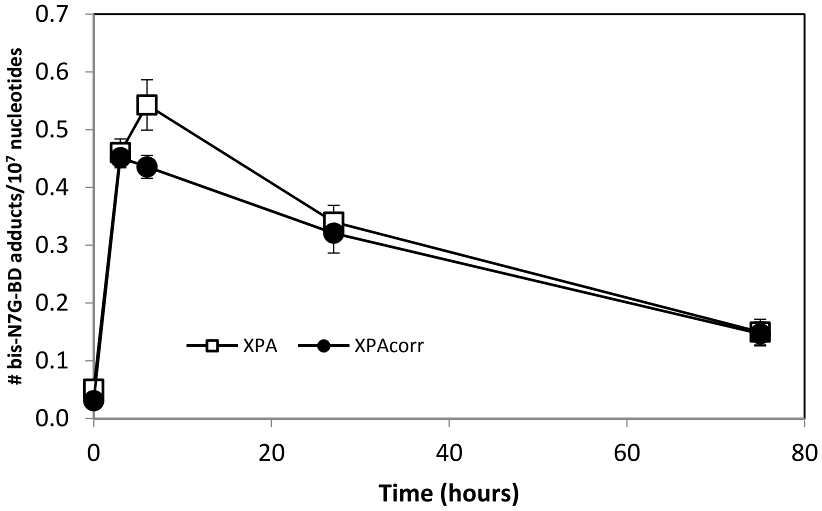

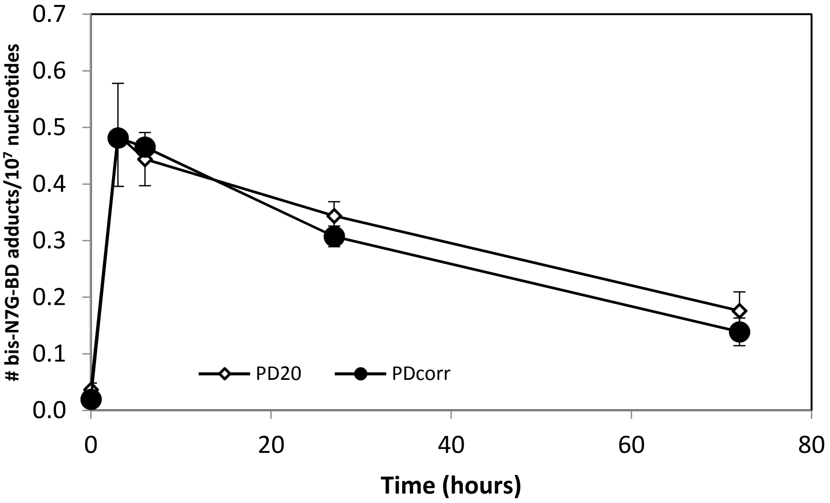

Figure S2. Time course for the formation and removal of *bis*-N7G-BD cross-links in human wild-type (circles), XPA (squares), and cells PD20 (diamonds). Cells were exposed to 15 μM DEB for 3 hours and then replaced with fresh growth media. Chromosomal DNA was isolated at the indicated times and b*is*-N7G-BD adduct levels were quantified by HPLC-ESI$^+$-MS/MS as described in the Methods section. Results represent average ± the SEM, N=3.
